# Subacromial impingement syndrome: association of multiple magnetic resonance imaging parameters with shoulder function and pain

**DOI:** 10.1007/s00402-021-04032-6

**Published:** 2021-07-06

**Authors:** Malte Jäschke, Hans-Christian Köhler, Marc-André Weber, Thomas Tischer, Claudia Hacke, Christoph Schulze

**Affiliations:** 1 Department of Diagnostic and Interventional Radiology, Pediatric Radiology and Neuroradiology, Rostock University Medical Center, Ernst-Heydemann-Str. 6, 18057 Rostock, Germany; 2 Department of Orthopaedics, Rostock University Medical Center, Doberaner Str. 142, 18057 Rostock, Germany; 3 Department of Trauma Surgery and Orthopaedics, German Armed Forces Hospital of Westerstede, Lange Str. 38, 26655 Westerstede, Germany; 4 Department of Pediatrics I, University Medical Center Schleswig-Holstein, Arnold-Heller- Straße 3, 24105 Kiel, Germany

**Keywords:** Subacromial impingement, MRI, Pain, Shoulder function, Constant Score, Critical shoulder angle

## Abstract

**Introduction:**

Shoulder pain is one of the most common complaints in orthopaedics. This study focusses on the relationship between shoulder function in subacromial impingement syndrome and imaging criteria in magnetic resonance imaging (MRI).

**Materials and methods:**

This prospective clinical trial included 69 patients treated for subacromial impingement syndrome. Shoulder function (Constant Score, range of abduction, abduction force) and pain were correlated with the following MRI parameters: tendinosis of the rotator cuff, “halo-sign” around the biceps tendon, subacromial distance, critical shoulder angle, size of subacromial osteophytic spurs and maximum width of subacromial and subdeltoid bursa. Statistical analyses included Pearson’s and Spearman’s coefficients of correlation, multiple regression analysis and Student’s *t*-test.

**Results:**

The Constant Score was correlated positively with the critical shoulder angle (*r* = 0.313; *p* = 0.009) and inversely with a “halo-sign” around the biceps tendon (rho =  −0.384; *p* = 0.001). There was no significant correlation between spur size and shoulder function, but the size of the subacromial and subdeltoid bursae was positively correlated with the subacromial spur’s size (subacromial bursa: coronal plane: *r* = 0.327; *p* = 0.006; sagittal view: *r* = 0.305; *p* = 0.011; subdeltoid bursa coronal view: *r* = 0.333 *p* = 0.005). The width of the subdeltoid bursa in coronal plane was positively correlated with shoulder pain (*r* = 0.248; *p* = 0.004) and negatively with the range of abduction (*r* =  −0.270; *p* = 0.025), as well as the mean (*r* =  −0.332; *p* = 0.005) and maximum (*r* =  −0.334; *p* = 0.005) abduction force.

**Conclusions:**

Shoulder function and pain in subacromial impingement are best predicted by the width of the subdeltoid bursa measured in the coronal MRI plane as an indicator of bursitis as well as the presence of a “halo-sign” around the biceps tendon indicating glenohumeral joint effusion. Presence of a subacromial spur could lead to subacromial and subdeltoid bursitis, which impairs shoulder function. Shoulder function seems not to be compromised by the presence of a subacromial spur in absence of bursitis.

This study was registered at the German Clinical Trials Register on 08 February 2013 (ID: DRKS00011548).

## Introduction

In orthopaedic practice shoulder pain is one of the most common complaints and it is often caused by impingement syndrome of the shoulder [1, 2]. While first described by Neer in 1972 [3], today different types of impingement syndrome of the shoulder are differentiated regarding the cause: primary and secondary extrinsic (outlet) impingement, intrinsic (non-outlet) impingement and internal impingement [4]. Subacromial impingement is the most common form of primary extrinsic impingement syndrome, while subcoracoidal impingement syndrome is less common [1]. Primary extrinsic impingement syndrome can be caused by different pathologies like a hooked or laterally downsloping acromion, subacromial bony osteophytic spurs, hypertrophy of the coracoclavicular ligament, an os acromiale or hypertrophic osteoarthritis of the acromioclavicular joint [4, 5]. It remains controversial whether intrinsic degeneration or external mechanical stress is responsible for rotator cuff disease and impingement syndrome [6, 7]. Due to different opinions on pathogenesis of shoulder impingement syndrome, conservative and operative therapy procedures were successfully performed. Both kinds of therapy lead to similar functional results one year after intervention, although conservative therapy leads to earlier return to work [8].

Imaging in impingement syndrome usually relies on different imaging modalities [9]. X-ray, magnetic resonance imaging (MRI) and ultrasound are most commonly used and MRI is considered the most reliable imaging modality for evaluation of the rotator cuff by many authors as it allows for evaluation of soft tissue as well as bony abnormalities like subacromial osteophytic spurs and acromioclavicular joint capsular hypertrophy [9, 10]. MRI allows for diagnosis of rotator cuff tears with greater inter-observer reliability in assessment of tear size, retraction status and atrophy compared to ultrasound in which tear size tends to be underestimated [11, 12]. Computed tomography (CT) can be added to further examine bony changes and CT-arthrography can be an alternative for patients with contraindications for MRI as it facilitates assessment of the labrum and cartilage [9]. MRI in patients with impingement syndrome should include proton-density weighted and T1-weighted images in a coronal plane and T2-weighted images in a sagittal plane, these sequences are usually incorporated in a standard examination protocol of the shoulder [9]. Direct MR-arthrography with intraarticular injection of a contrast agent can be used to further evaluate the labrum as well as cartilage and allows better identification of subtle rotator cuff abnormalities than conventional MRI [13].

However, the influence of pathological findings in MRI on the extent of symptoms and functional impairment presented by these patients is not finally characterised. While many attempts across different imaging modalities have been made to find correlation between rotator cuff pathologies and morphologic changes like the form of the acromion, the coracoacromial arch, subacromial spurs or the narrowing of the subacromial space [6, 10, 14–18] this study focusses on the relationship between shoulder function and imaging criteria in patients with subacromial impingement syndrome.

## Materials and methods

### Study subjects

106 patients that were treated for subacromial shoulder impingement between July 2013 and June 2017 at the Department of Trauma Surgery and Orthopaedics at the German Armed Forces Hospital of Westerstede were enrolled in this prospective clinical study. Inclusion criteria were clinical signs of isolated subacromial impingement syndrome (at least one of the following test with a positive result: painful arc, impingement tests according to Neer or Kennedy–Hawkins), shoulder pain for at least six weeks, a diagnostic MRI scan performed within 100 days pre- and post-first consultation and before initiation of treatment, patient age ≥ 18 years and ≤ 70 years and written informed consent. Before inclusion in this study, patients had received non-standardised conservative care of varying amount by different referring physicians with no sufficient improvement of symptoms for at least six weeks.

**Fig. 1:**
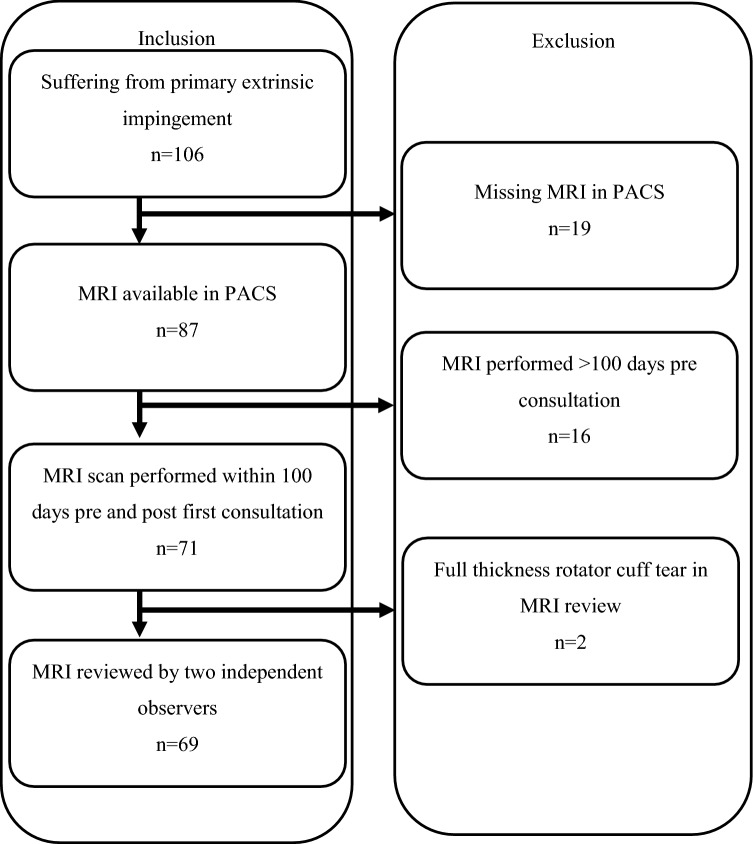
The procedure for inclusion is demonstrated here. *MRI* magnetic resonance imaging; *PACS* picture archiving and communication system

Exclusion criteria were presence of a rheumatic disease, symptomatic osteoarthritis of the shoulder, shoulder instability, pathologies of the tendon of the long head of the biceps, injuries to the glenoid and partial tears greater than 3 mm as well as full thickness tears of the rotator cuff. Figure 1 shows the sequence of inclusion. In 16 patients the MRI scan was older than 100 days at the start of our study. These patients were excluded from further analysis. During evaluation of these data we observed two patients with full thickness tears of the rotator cuff. Accordingly, 69 patients were considered in the final analysis.

The following data were assessed: biometric data (e.g. height, weight, age, and sex), shoulder function (Constant Score, range of abduction, abduction force) and pain using the Numerical Rating Scale (NRS). Patient characteristics are given in Table 1. For the measurement of the mean and maximum abduction force the patient had to pull an isometric dynamometer (IsoForceControl EVO2, Fa. MDS, Oberburg, Switzerland) with maximum force for 10 s in a 90° position with laterally abducted arm.

**Table 1:** Patient characteristics

Variable	*n* = 69
Age (years)	46.23 ± 11.50
Number of females	28 (40.6%)
Body mass index (kg/m^2^)	28.47 ± 5.74
Height (m)	1.76 ± 0.09
Weight (kg)	88.05 ± 17.18

Values are means and standard deviations or proportions

The responsible local ethics commission approved the study (Ref.: A-2013-0135).

### MRI parameters

All MRI scans were reviewed by two independent observers, who were blinded to the clinical data of the patients. One observer was a consultant orthopaedic shoulder specialist, the other observer a radiologist working in a university hospital specialised in musculoskeletal imaging.

MRI scans were performed in outpatient care. In six patients (8.7%) MRI was performed shortly after first consultation. Each MRI scan contained at least one transversal, sagittal and coronal plane consisting of at least one T1-weighted and at least one proton density (PD)-weighted fat saturated or T2-weighted sequence.

The following parameters were assessed: presence of a “halo-sign” around the tendon of the long head of the biceps muscle, which correlates with fluid in the synovial sheath indicating effusion in the glenohumeral joint [19]. It was rated positive if a closed hyperintense circle around the tendon of the long head of the biceps muscle was present in at least one imaging plane of a transversal PD-weighted fat saturated or T2-weighted sequence.

**Fig. 2:**
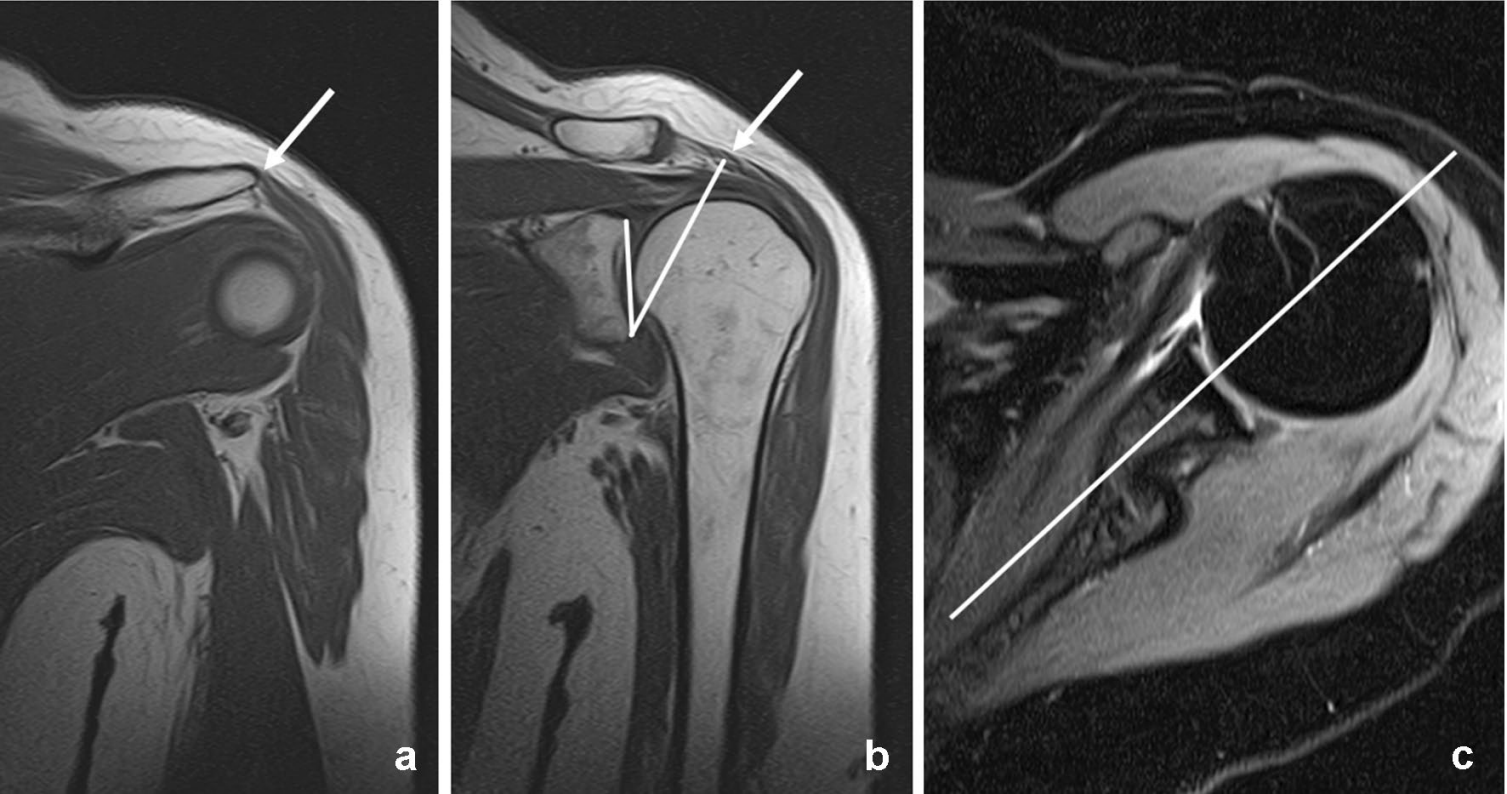
Measurement of the critical shoulder angle. T1-weighted images in a coronal plane **a**, **b**. Transversal proton-density weighted fat saturated images **c**. The most lateral extent of the acromion is marked in a coronal plane (*arrow* in **a**). After scrolling to a coronal plane, representing the middle of the glenoid **b**, the critical shoulder angle was measured. The white line in **c** represents the imaging plane of **b**

The measurement of the critical shoulder angle (CSA) was adapted to MRI as previously described by Spiegl et al. [20]. As the most lateral point of the acromion is usually located posteriorly to the glenoid the most lateral extent of the acromion in a coronal plane is marked with the cursor (see Fig. 2). After scrolling back to a plane that shows the glenoid and more central parts of the acromion, the critical shoulder angle was measured [20].

Tendinosis of the supraspinatus muscle, infraspinatus muscle and subscapularis muscle was graded in a three-point scale according to Bauer et al. [21]. A uniform low signal of the tendon in PD-weighted fat saturated or T2-weighted sequences was scored as “Grade 0”, increased but not fluid signal in these sequences extending not more than 10 mm in any dimension as “Grade 1” and if larger than 10 mm in any direction as “Grade 2” [21].

If present, partial rotator cuff tears were noted. If the two readers disagreed on the presence of a tear, these cases were judged by a third reader. The third reader was a senior consultant orthopaedic shoulder specialist with more than 15 years of experience in reading shoulder MRI who was also blinded to all clinical information of the cases.

The minimal distance between the humeral head and the acromion was measured in a coronal and sagittal plane.

**Fig. 3:**
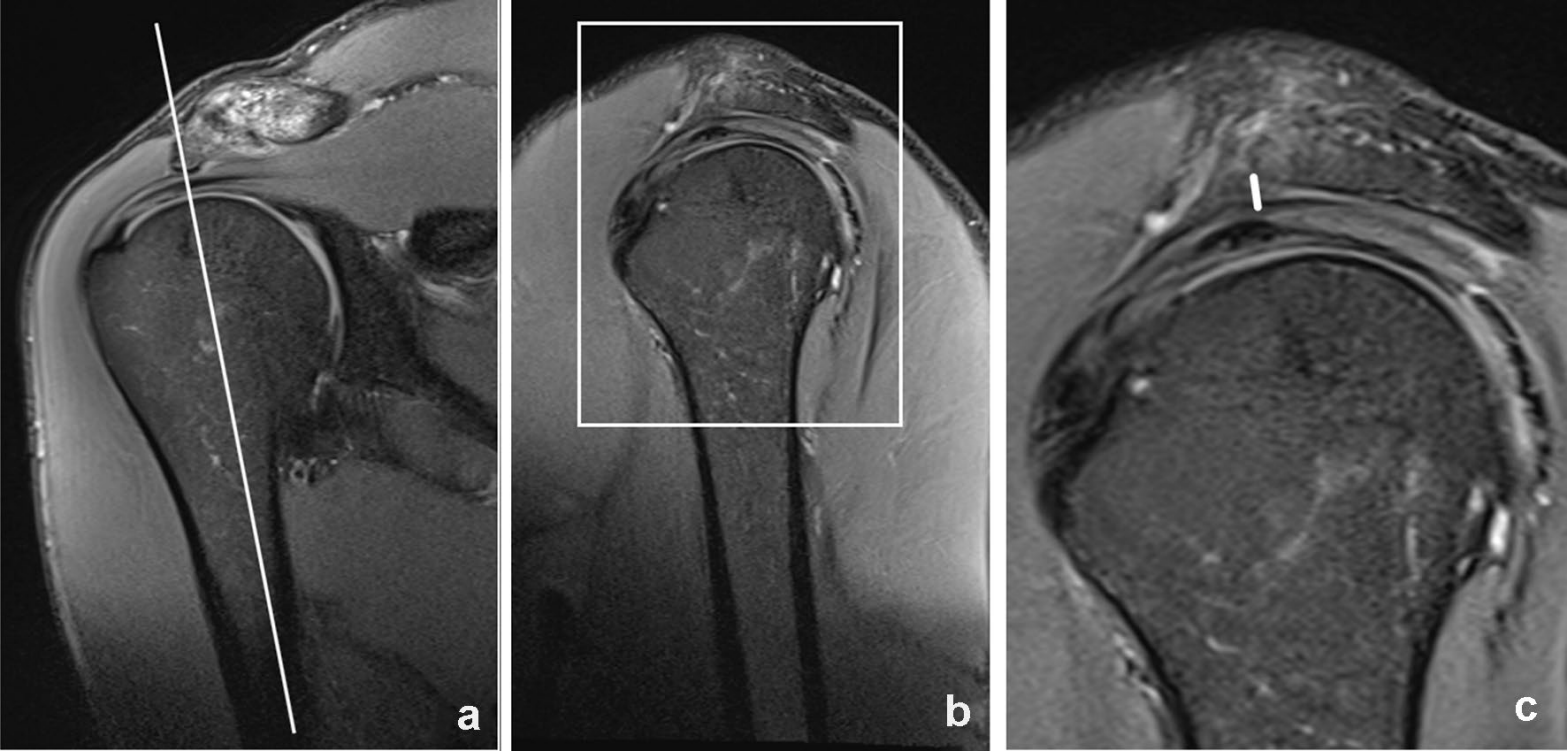
Measurement of a subacromial spur. Proton-density weighted fat saturated images in a coronal **a** and sagittal **b**, **c** plane. The size of the subacromial spur was measured in a sagittal plane according to the axis of the humeral head, the measured length is represented by a white line in **c**. The rectangle in **b** represents the area which is enlarged in **c**. The white line in **a** shows the imaging plane of the sagittal images **b**, **c**

**Fig. 4:**
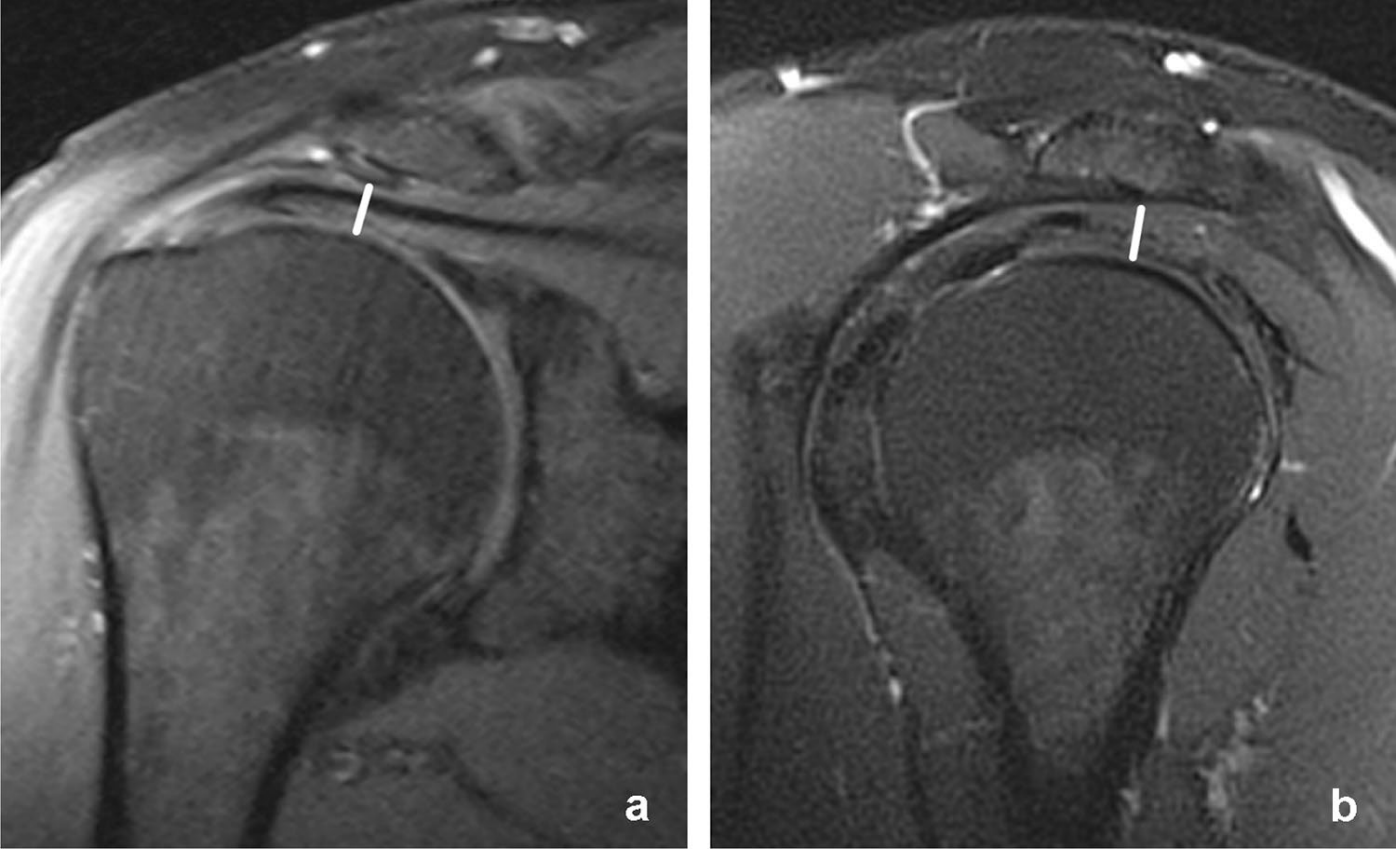
Measurement of the subacromial distance. Proton-density weighted fat saturated images in a coronal **a** and sagittal **b** plane. The minimum distance between the acromion and the head of the humerus was measured in both planes. In this case the subacromial space was narrowed by a subacromial spur

If a subacromial osteophytic spur was present, it was measured in a sagittal plane (see Fig. 3) and the minimal distance between the caudal border of the spur instead of the caudal margin of the acromion itself was referred to when measuring distance to the humeral head (see Fig. 4).

The maximum width of both the subacromial and the subdeltoid bursa were measured in a sagittal MRI plane for the ventrodorsal dimension and in a coronal plane for the left-to-right dimension.

All MRI parameters were assessed electronically using a PACS workstation (Carestream Vue PACS Version 11.4.1.1011, Carestream, Rochester, New York, USA).

### Statistical analysis

For descriptive purposes, continuous data are expressed as the mean ± standard deviation (SD), and categorical variables were described using absolute/relative frequency distribution. Data were analysed by bivariate correlation analysis using the Pearson coefficient (r) to evaluate simple associations between continuous parameters. Spearman’s correlation (rho) coefficient was performed to assess correlation between ordinal variables. Correlation coefficients were interpreted according to Zou et al. [22] as weak, moderate, strong and perfect. Multiple linear and ordinal logistic regression models were subsequently used to determine whether critical shoulder angle, “halo-sign”, bursitis and rotator cuff changes were associated with Constant Score, pain and range of motion. Interrater agreement was analysed as proportion of agreement. Interrater reliability assessment was performed using kappa statistics, intraclass correlation coefficient (ICC) and kendalls tau (*Ƭ*_b_). Normality of data distribution was verified using the Shapiro–Wilk test. Two-tailed Student’s *t*-test was used to evaluate differences in two independent groups. A *p*-value ≤ 0.05 was considered to indicate statistical significance. All statistical analyses were performed with IBM SPSS Statistics 23.0 (SPSS, Chicago, Illinois, USA) by an independent statistician.

## Results

### Shoulder examination

Mean shoulder pain was (NRS) 5.61 ± 1.53 [1–8] and maximum abduction was 133.26 ± 41.37° [30–180°]. Strength testing showed maximum abduction force of 148.20 ± 100.65 N [17.0–385.5 N] and mean abduction force of 118.17 ± 80.98 N [14.2–313.4 N]. The mean Constant Score was 56.03 ± 17.15 [20–87].

### Critical shoulder angle

The inter-observer variability for the measurement of the critical shoulder angle was very good (ICC = 0.943; *p* < 0.001). Mean critical shoulder angle was 31.2 ± 4.9° [15.5–45.5°]. The critical shoulder angle was positively correlated with the Constant Score (*r* = 0.313; *p* = 0.009), the width of the subacromial bursa measured in a coronal (*r* = 0.422; *p* = 0.005), and the sagittal plane (*r* = 0.305; *p* = 0.004). There was no significant correlation between CSA and level of pain (*r* =  −0.148; *p* = 0.226). In the correlation and regression analysis, the variance in the Constant Score was significantly predicted by the MRI-based parameters critical shoulder angle (*p* = 0.009), width of subdeltoid bursa (*p* = 0.050; see Table 2), and “halo-sign” (*p* = 0.001; see Table 3).

**Table 2:** Results of the multiple linear regression analysis

	*b* (SE)	95% CI	beta	*p*	*R*^2^
Model constant score					
CSA	0.092 (0.034)	21.839; 29.826	0.313	**0.009**	0.098
B. subdeltoidea, coronal	0.014 (0.007)	− 0.027; 0.000	− 0.237	**0.050**	0.056
Model maximum abduction force					
B. subacromialis, coronal	− 0.003 (0.001)	− 0.05; − 0.001	− 0.311	**0.009**	0.097
B. subacromialis, sagittal	− 0.020 (0.001)	− 0.04; 0.000	− 0.223	0.065	0.05
B. subdeltoidea, coronal	− 0.003 (0.001)	− 0.006; − 0.001	− 0.334	**0.005**	0.112
Model mean abduction force					
B. subacromialis, coronal	0.040 (0.001)	− 0.07; − 0.001	− 0.340	**0.004**	0.116
B. subacromialis, sagittal	− 0.003 (0.001)	− 0.06; 0.000	− 0.253	**0.036**	0.640
B. subdeltoidea, coronal	− 0.004 (0.001)	− 0.007; − 0.001	− 0.332	**0.005**	0.110
Model shoulder pain					
B. subdeltoidea, coronal	0.160 (0.076)	0.008; 0.312	0.248	**0.040**	0.061
B. subdeltoidea, sagittal	0.242 (0.085)	0.073; 0.412	0.330	**0.006**	0.109
Model range of abduction					
B. subdeltoidea, coronal	− 0.006 (0.003)	− 0.012; − 0.001	− 0.270	**0.025**	0.073

Significant *p*-values are given in bold

*b* unstandardized regression coefficient; *SE* standard error; *95% CI* 95% confidence interval; *beta* standardised regression coefficient; *R*^*2*^ determination coefficient, *CSA* critical shoulder angle; *B. subdeltoidea* bursa subdeltoidea; *B. subacromialis* bursa subacromialis. Coronal and sagittal refers to the plane in which the width of each bursa was measured

**Table 3:** Results of the ordinal regression analysis

	Regression coefficient (SE)	95% CI	*p*
Model constant score			
“Halo-sign” biceps	− 0.053 (0.017)	− 0.086; − 0.021	**0.001**
Model maximum abduction force			
“Halo-sign” biceps	− 0.008 (0.003)	− 0.014; − 0.003	**0.002**
Model mean abduction force			
M. supraspinatus tendinosis	− 0.008 (0.003)	− 0.014; − 0.002	**0.007**
Model range of abduction			
“ Halo-sign” biceps	− 0.16 (0.06)	− 0.28; − 0.003	**0.013**

Significant *p*-values are given in bold

*SE* standard error, *95% CI* 95% confidence interval

### Rotator cuff

Grading of tendinosis showed strong interrater variability for the supraspinatus muscle (*Ƭ*_b_ = 0.877; *p* < 0.001), the subscapularis muscle (*Ƭ*_b_ = 0.893; *p* < 0.001) and the infraspinatus muscle (*Ƭ*_b_ = 1; *p* < 0.001). Mean tendinosis grade was 0.80 ± 0.66 [0–2] for the supraspinatus muscle, 0.20 ± 0.48 [0–2] for the infraspinatus muscle, and 0.23 ± 0.49 [0–2] for the subscapularis muscle. Tendinosis of the supraspinatus muscle (*n* = 45) was negatively correlated with the average abduction force (rho =  −0.295; *p* = 0.014).

There was no significant correlation observed between tendinosis of the supraspinatus respective infraspinatus muscle and the Constant Score, shoulder pain and the range of abduction. But correlation and regression analysis showed that the variance in the mean abduction force (*p* = 0.007) was predicted to a significant extent by the variable tendinosis of the supraspinatus muscle (see Table 3).

The inter-observer variability for the presence of partial tears of the rotor cuff was almost perfect for the supraspinatus muscle (κ = 0.925; *p* < 0.001), moderate for the infraspinatus muscle (κ = 0.553; both *p* < 0.001), and perfect for the subscapularis muscle (κ = 1; *p* < 0.001). Eighteen patients (26.1%) had tendinosis and a minimal partial tear (smaller than 3 mm) of the supraspinatus tendon, one patient (each 1.45%) had a partial tear of the subscapularis and infraspinatus muscle, respectively.

### “Halo-sign” around the long head of the biceps muscle

The interobserver variability for the presence of a “halo-sign” around the long head of the biceps muscle was almost perfect (κ = 0.82; *p* < 0.001). The “halo-sign” was present in 31 patients (36.5%). In seven patients, the two readers disagreed on the presence of a “halo-sign”. These patients were considered separately in further statistical analysis. The “halo-sign” was negatively correlated with the Constant Score (rho =  −0.384; *p* = 0.001), the range of maximum abduction (rho =  − 0.268; *p* = 0.026), the average abduction force (rho =  −0.324; *p* = 0.007) and the maximum abduction force (rho =  −0.349; *p* = 0.003). The correlation and regression analysis demonstrated that the variance in the maximum abduction force (*p* = 0.002), the Constant Score (*p* = 0.001) and range of abduction (*p* = 0.013) were predicted to a significant extent by the variable “halo-sign” (see Table 3).

### Subacromial spur and distance between acromion and head of the humerus

The inter-observer variability for the size of a subacromial spur was nearly perfect (ICC = 0.968; *p* < 0.001). A subacromial spur was present in 56 patients (81%) with a mean size of 1.28 ± 1.12 mm [0–4 mm]. The size of the spur was positively correlated with the width of the subacromial bursa in both imaging planes (coronal: *r* = 0.327; *p* = 0.006; sagittal: *r* = 0.305; *p* = 0.011) and the width of the subdeltoid bursa in coronal plane (*r* = 0.333; *p* = 0.005). There was no significant correlation between spur size and shoulder function or pain.

The inter-observer variability for the measurement of the minimum distance between the acromion and head of the humerus was very good in a coronal (ICC = 0.957; *p* < 0.001) as well as a sagittal MRI plane (ICC = 0.929; *p* < 0.001). Correlation between measurements in both planes was moderate (*r* = 0.79; *p* < 0.001). Mean distance was 10.08 ± 1.94 mm [6.0–16.0 mm] in a coronal plane and 10.36 ± 1.82 mm [7.0–14.5 mm] in a sagittal plane. Both measurements were significantly and inversely correlated with the size of a subacromial spur (coronal: *r* =  −0.316; *p* = 0.008; sagittal: *r* =  −0.270; *p* = 0.025), but there was no significant correlation with shoulder function or pain.

### Bursa subacromialis and subdeltoidea

The inter-observer variability for the maximum width of the subacromial and subdeltoid bursa was very strong in a coronal (subdeltoid bursa: ICC = 0.921; *p* < 0.001; subacromial bursa: ICC = 0.978; *p* < 0.001) and sagittal plane (subdeltoid bursa: ICC = 0.942; *p* < 0.001; subacromial bursa: ICC = 0.949; *p* < 0.001).

The width of the subacromial bursa measured in a coronal plane was negatively correlated with the mean (*r* =  −0.340; *p* = 0.004) and maximum abduction force (*r* =  −0.311; *p* = 0.009). The mean abduction force was negatively correlated with the width of the subacromial bursa measured in a sagittal plane (*r* =  −0.253; *p* = 0.036).

The width of the subdeltoid bursa measured in a coronal plane was positively correlated with shoulder pain (*r* = 0.248; *p* = 0.004), negatively with range of abduction (*r* =  −0.27; *p* = 0.025), mean (*r* =  −0.332; *p* = 0.005) and maximum abduction force (*r* =  −0.334; *p* = 0.005). When measured in a sagittal plane, a positive correlation was observed only with shoulder pain (*r* = 0.330; *p* = 0.006) but not with range of abduction, mean and maximum abduction force.

## Discussion

The most important results of our study are the positive correlation between the extent of bursitis and functional deterioration and that a subacromial spur alone (without bursitis) was not significantly correlated with functional deterioration or pain level.

Although involving scrolling through different imaging planes (see Fig. 2), adaption of the critical shoulder angle from conventional radiographs to MRI is a reliable measurement with almost perfect interrater variability (κ = 0.829; *p* < 0.001). This is remarkable since other authors only reported an inter-observer agreement of 0.62 for their method of measuring the critical shoulder angle [20]. One reason for this might be that other study populations consisted to one third of patients with osteoarthritis of the glenohumeral joint making it hard to identify the cranial and caudal border of the glenoid, which is needed for measuring the critical shoulder angle [20]. So the accuracy of measuring CSA in Impingement/rotator cuff populations seems to be more valid than in case of other pathologies.

A high critical shoulder angle in radiography is a known risk factor for outlet impingement [1]. Critical shoulder angle values greater than 35° are supposed to be associated with rotator cuff tears and lower than 30° with glenohumeral osteoarthritis [23, 24]. While checking this hypothesis was not part of our study, we could demonstrate a significant positive correlation of the critical shoulder angle with the width of the subacromial bursa. As the width of the bursa is determined by the amount of fluid it contains, larger measurements were interpreted as a sign of bursitis. Subacromial bursitis is a hallmark of subacromial impingement, which is often observed in coincidence with rotator cuff tears. Thus, our finding might support the previously stated hypothesis that higher critical shoulder angles are supposed to be associated with rotator cuff tears.

Since our study included patients with primary extrinsic shoulder impingement, the significant positive correlation of the critical shoulder angle with the Constant Score indicates that higher CSA values seem to have no negative effects on shoulder function in these patients. One limitation are the low correlation coefficients. However, regression analysis supported the hypothesis, that the critical shoulder angle is positively correlated with the Constant Score. Our statistical analysis showed that pain tended to be negatively correlated with CSA. So, it seems to be a fact, that CSA in MRI cannot be used to explain low levels of function but can be a predictor for higher levels of pain in case of subacromial impingement of the shoulder. There is a strong association between CSA and subacromial and subdeltoid bursitis. It should be pointed out that mean critical shoulder angle (31.2 ± 4.9°) was not greater than 35°, as reported in radiographic studies as a cutoff value [23], although we only included patients with primary extrinsic shoulder impingement which certainly is closer related to rotator cuff pathology than glenohumeral osteoarthritis. The relation between CSA in MRI and radiographic imaging was not checked in this study, which is a limitation.

The presence of a “halo-sign” around the long head of the biceps muscle correlated significantly and inversely with the Constant Score, the range of maximum abduction as well as the mean and maximum abduction force. Park et al. previously demonstrated significant negative correlation between the amount of joint effusion around the biceps tendon measured in ultrasound and different functional scores including the American Shoulder and Elbow Surgeons Score (*r* =  −0.400; *p* < 0.05) and the Simple Shoulder Test (*r* =  −0.275; *p* < 0.05) [25]. It can be concluded that the study by Park et al. as well as our current study show the connection of joint effusion and deteriorated shoulder function. Our results were supported in the regression analysis, which proved significant influence of the presence of a “halo-sign” on maximum abduction force, Constant Score and range of abduction.

While we are not aware of a validated definition of the “halo-sign” in MRI, our method of rating this sign (closed hyperintense circle around the biceps tendon in at least one imaging plane of a transversal PD-weighted or T2-weighted sequence) proved as a reliable scoring method with almost perfect interrater variability. It was only rated whether the “halo-sign” was present, but the amount of fluid around the biceps tendon was not further quantified, which is another limitation of this study.

The M. supraspinatus showed strong interrater reliability regarding the presence of a minimal partial tear and in grading tendinosis. Bauer et al. [21] who first described the used grading system for tendinosis reported lower inter-observer agreement regarding the supraspinatus muscle (0.69) than we found (0.877).

Tendinosis of the supraspinatus muscle was significantly inversely correlated with the mean abduction force matching the function of the supraspinatus muscle. Although the correlation coefficient was weak, the result was supported by regression analysis. It must be pointed out that there was no significant correlation between supraspinatus tendinosis and the Constant Score, shoulder pain and the range of abduction. However, although not significant, negative correlation between supraspinatus tendinosis and the Constant Score and range of abduction was observed. This trend supported the significant results regarding the abduction force and the number of cases might be the limitation here.

Shoulder pain had the strongest correlation with the width of the subdeltoid bursa measured in a coronal plane. Therefore, the width of the subdeltoid bursa measured in a coronal plane seems to be a feasible parameter to assess shoulder function in patients with primary extrinsic shoulder impingement. This hypothesis is further supported by the regression analysis in which the width of the subdeltoid bursa measured in a coronal plane was the only MR imaging parameter that could significantly predict the Constant Score, mean and maximum abduction force, shoulder pain and the range of abduction (see Table 2). This is not surprising since the maximum width of each bursa can be interpreted as an indicator of bursitis. A possible connection of both bursae, whose presence is still in debate [26], could explain the significant correlation of the maximum width of both bursae. Another explanation could be that bursitis of both bursae seems to occur together.

The width of both bursae was significantly correlated with the size of a subacromial spur, which is remarkable since the presence of a subacromial spur is thought to be a risk factor for subacromial impingement syndrome [1]. However, spur size did not correlate significantly with shoulder function and pain which is supported by findings that subacromial decompression has no benefit in functional outcome and pain [27]. Our findings suggest that the presence of a subacromial spur can lead to subacromial and subdeltoid bursitis, which causes deteriorated shoulder function. Shoulder function seems not be compromised by the presence of a subacromial spur in absence of bursitis.

In a metanalysis, including 18 studies, McCreesh et al. showed that MRI (as well as CT) provide more reliable measurements of the acromiohumeral distance than conventional radiographs [28]. This meta-analysis also included a study by Saupe et al. that demonstrated significant correlation between tendon tears and fatty muscle degeneration with reduced acromiohumeral distance [29]. Saupe et al. also showed high interrater reliability for evaluation of the acromiohumeral distance (0.97–0.99) which was measured in sagittal plane [29]. In our study interrater reliability for measuring the evaluation of the acromiohumeral distance was also very good supporting the hypothesis that MRI provides reliable measurement of the acromiohumeral distance.

Unsurprisingly, measurements in both planes correlated inversely with the size of a subacromial spur. We did not observe any significant correlation between shoulder function (range of abduction, abduction force, Constant Score) and acromiohumeral distance.

One general limitation of our study is that all MRI scans were carried out in outpatient care without standardised radiological examination protocol and varying time between study enrollment, MR imaging and assessment of clinical data. In most cases the MRI was performed before but sometimes after clinical assessment. Changes in structural conditions may be possible in the time span between clinical assessment and MRI. To limit the extend of influence, only patients with a MRI scan performed within 100 days pre and post first consultation and before initiation of treatment were considered in final analysis.

## Conclusion

Shoulder function and pain in subacromial impingement are best predicted by the width of the subdeltoid bursa measured in a coronal MRI plane as an indicator of bursitis and the presence of a “halo-sign” around the biceps tendon indicating glenohumeral joint effusion. Critical shoulder angle detected with the described method significantly influences shoulder function and correlates positively with the width of the subacromial bursa. Our findings suggest that the presence of a subacromial spur leads to subacromial and subdeltoid bursitis, which causes deteriorated shoulder function. Shoulder function seems not to be compromised by the presence of a subacromial spur in absence of bursitis.
